# Radial Artery Occlusion Impairs Median Nerve Perfusion—A Study Using Microvascular Imaging in Healthy Volunteers

**DOI:** 10.3390/diagnostics16050695

**Published:** 2026-02-27

**Authors:** Tobias Rossmann, Paata Pruidze, Johannes Mayerhofer, Michael Veldeman, Wolfgang K. Pfisterer, Wolfgang J. Weninger, Stefan Meng

**Affiliations:** 1 Division of Anatomy, Medical University of Vienna, Waehringer Strasse 13, 1090 Vienna, Austria; 2 Department of Neurosurgery, Neuromed Campus, Kepler University Hospital, 4020 Linz, Austria; 3 Clinical Research Institute for Neurosciences, Faculty of Medicine, Johannes Kepler University Linz, 4020 Linz, Austria; 4 Department of Radiology, Hanusch Hospital, 1140 Vienna, Austria; 5 Department of Neurosurgery, RWTH Aachen University Hospital, 52074 Aachen, Germany; 6 Department of Neurosurgery, Clinic Donaustadt, 1220 Vienna, Austria

**Keywords:** transradial, access, Allen, Barbeau, color Doppler ultrasound, carpal tunnel syndrome

## Abstract

**Background/Objectives:** The transradial approach is widely used for vascular access in many disciplines. Radial artery occlusion (RAO) is a frequent sequel, and hand/arm pain affects 7.8% of patients. We aimed to elucidate whether RAO or ulnar artery occlusion (UAO) causes impaired neural blood flow and, thus, if symptoms may be attributable to claudication or nerve damage. **Methods:** Forty healthy volunteers (73% female), with a mean age of 38 years and without clinical or sonographic signs of carpal tunnel syndrome, were included. All underwent a standardized ultrasound examination (Aplio i800 and i22LH8 linear transducer, Canon Medical Systems) of the forearm, investigating the median nerve and its intraneural blood flow as well as the vascular status of the limb. The radial and ulnar arteries were then sequentially compressed, while changes to intraneural blood flow were noted. Thereafter, the (reverse) Barbeau test and the (inverse) modified Allen Test (MAT) were performed. **Results:** Simulated RAO and UAO halted intraneural blood flow in 65% and 62.5% of individuals, respectively. A total of 32.5% of participants reported discomfort in the hand/arm. Absent flow during occlusion was found at a significantly higher rate in symptomatic individuals. MAT and inverse MAT were abnormal (>10 s) in 17.5% and 7.5% of patients. Barbeau and reverse Barbeau produced type D results in 15% and 20%, respectively. **Conclusions:** Both simulated RAO and UAO caused the cessation of intraneural blood flow of the median nerve in two-thirds of participants, and a large proportion reported symptoms. MAT and Barbeau tests did not seem to be useful in predicting impaired neural blood flow.

## 1. Introduction

Transradial access has become the standard access in percutaneous cardiovascular intervention [1,2] and shows encouraging results in peripheral endovascular procedures [3], but has not yet found widespread application in neurointervention despite its merits [4]. Radial artery occlusion (RAO) is a potential complication of arterial puncture in those procedures; rates of 0.2–3.6% have been estimated [1,3,5]. Although consecutive ischemic symptoms and irreversible structural damage to the hand are rare, the impairment of hand function and especially hand or arm pain are not infrequent [1,2,6]. The application of preventive measures has been emphasized [7], but practices to prevent RAO are not widely implemented in clinical practice [8]. Apart from the risk of irreversible structural damage to the upper limb, ischemia may also cause damage to various tissues, including peripheral nerves. Based on a recent survey [8], simple bedside tests such as the modified Allen Test (MAT) [9] or the Barbeau test [10] are still used in some settings, despite their low clinical value when predicting ischemia of the hand [11]. It remains unknown whether the MAT or Barbeau tests predict neural ischemia or related symptoms.

This study sets out to evaluate whether simulated RAO or ulnar artery occlusion (UAO) impairs intraneural blood flow of the median nerve. It is of interest if changes in neural blood flow correlate to symptoms and whether the MAT or Barbeau test can predict their occurrence.

## 2. Materials and Methods

### 2.1. Study Participants

The volunteers for this study were recruited via a public announcement at the Department of Radiology of the PI’s institution. All individuals aged 18 or above were invited to participate, provided that they had no medical history of nerve entrapment syndromes or of peripheral nerve disorders, especially carpal tunnel syndrome (CTS). Freedom of symptoms suggestive of CTS was assessed using the German translation [12] of the Boston Carpal Tunnel Questionnaire (BCTQ) [13]. Further exclusion criteria were pregnancy (ruled out via a urine HCG test), diabetes, rheumatoid arthritis, polymyalgia rheumatica, hypothyroidism, acromegaly, amyloidosis or carcinomatosis.

All eligible participants were screened bilaterally for detectable intraneural blood flow in the median nerve. Whether the left or right arm was to be investigated was randomly assigned using an online tool (Random Integer Generator, random.org). In case there was only unilateral flow, this side was used instead. In total, 52 individuals were screened until the pre-specified number of 40 participants was met; 12 (23%) showed no detectable intraneural blood flow.

### 2.2. Standardized Ultrasound Protocol

**Figure 1:**
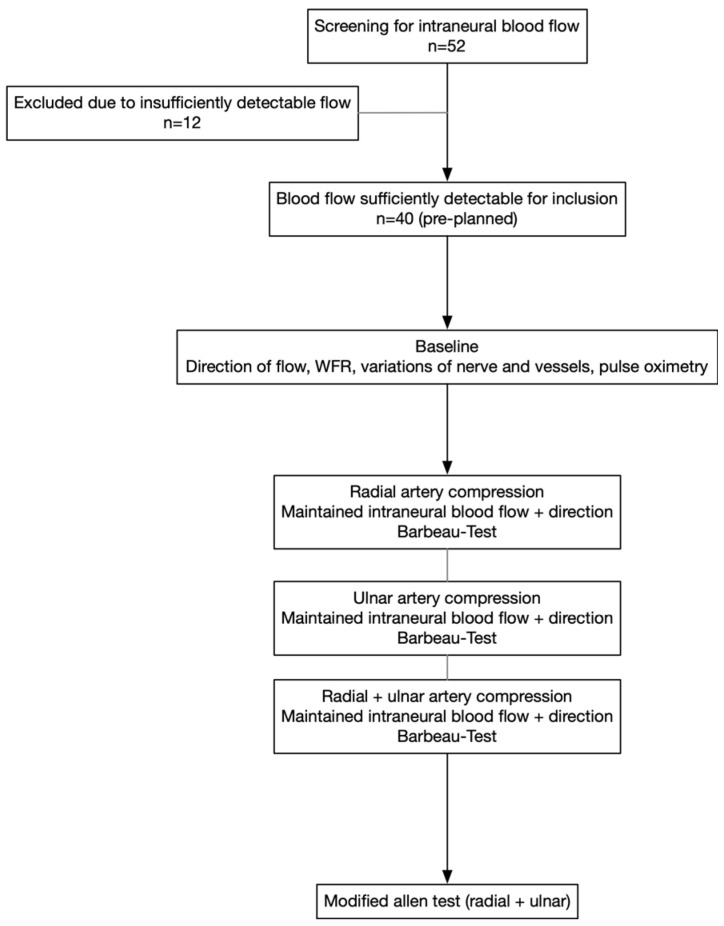
Flowchart of enrollment and investigations.

A flowchart of the course of the investigation is provided in Figure 1. The same high-resolution ultrasound system (Aplio i800 and i22LH8 linear transducer, Canon Medical Systems Europe B.V., Amstelveen, The Netherlands) was used in all participants; all exams were performed by the same radiologist experienced in peripheral nerve sonography.

Arms were placed supine in a neutral position, avoiding any hyperextension. As baseline measurements, the direction of intraneural blood flow was noted, and the median nerve was investigated from mid forearm to the palm. The wrist-to-forearm ratio (WFR) [14], any anatomical variations in nerve and blood vessels, any stenosis of the ulnar and radial artery and the existence of a superficial palmar arch were noted. A pulse oximeter (Checkme™ Pod, Viatom Technology Co., Ltd., Shenzhen, China) was placed on the middle finger, and the plethysmographic signal was observed until stable.

The radial artery was then compressed by an assistant proximal to the pronator quadratus; efficient compression of the vessel was confirmed by ultrasound. The median nerve at the carpal tunnel was then again scanned for intraneural blood flow and, if still present, the direction of flow.

### 2.3. Barbeau Test

In the meantime, for at least two minutes, the pulse oximeter was observed by an assistant to assign findings to one of the four categories described by Barbeau et al. [10]: (A) no damping of pulse wave immediately after compression of the radial artery; (B) damping of the pulse wave; (C) loss of pulse wave followed by recovery within 2 min; (D) loss of pulse wave without recovery within 2 min. This procedure was repeated with isolated compression of the ulnar artery (reverse Barbeau test) and then by simultaneously compressing both the ulnar and radial artery.

### 2.4. Modified Allen Test

The modified Allen Test was performed in a standard fashion. Both the radial and ulnar artery were compressed by the investigator, and the participant clenched his/her fist to squeeze out venous blood from the hand. Compression of the ulnar artery was then released, and the time to return of a full blush was measured to simulate radial artery occlusion. The test was repeated with a later release of the radial artery (inverse MAT). The findings were interpreted as previously described by others [15]; zero to five seconds were rated as normal, six to ten seconds as “intermediate”, and abnormal if exceeding ten seconds.

All compression tests were performed in direct consecutive order, without any delay or predefined intervals of recovery.

### 2.5. Statistics

Data were analyzed using SPSS Statistics 30.0 (IBM Inc., Armonk, NY, USA) for descriptive statistical analysis. Normality of data was tested using the Shapiro–Wilk test; normally distributed data are presented as mean (standard deviation) or otherwise as median (interquartile range, IQR). Comparison of metric variables was performed using the Mann–Whitney U-test due to small group sizes; comparison of categorical variables was performed using Chi-squared or Fisher’s exact test. A two-sided *p*-value of <0.05 was considered statistically significant.

## 3. Results

The 40 participants (77% of screened individuals) that met the inclusion criteria had a mean age of 38 years, and 73% were female. The examination of left and right arms was almost balanced, being the dominant hand in 48% of cases. No anatomical variations in the median nerve were present; the median WFR was 1.444. The full participant characteristics are presented in Table 1. BCTQ was without any pathological findings in all participants, except for two individuals with a mean symptom severity scale of 1.18.

### 3.1. Intraneural Flow During Arterial Compression

**Figure 2:**
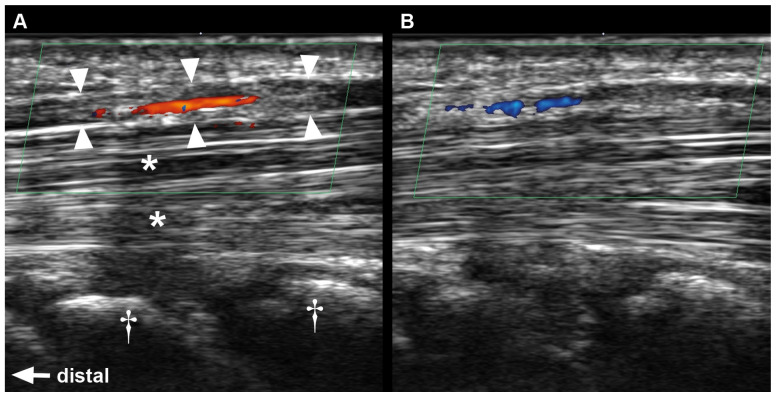
Intraneural blood flow reversal during compression of the ulnar artery. (**A**) Intraneural blood flow at baseline. (**B**) Same nerve segment during compression of the ulnar artery. Arrowheads outline median nerve. Asterisks = flexor tendons. Daggers = carpal bones.

At baseline, the direction of blood flow in the median nerve at the carpal tunnel was antegrade (from proximal to distal) in 95% of cases, while being retrograde in two individuals (5%). During the compression of the radial artery, flow was only visible in 14 individuals (35%), still antegrade in the majority of cases (93%). The reversal of flow direction occurred only in one participant (Table 2). Likewise, during ulnar artery compression, blood flow was detectable in only 38% (15 individuals). Flow was antegrade in 80% and retrograde in 20%, and flow reversal was found in two cases (13%). Sonographic images of one of those cases are shown in Figure 2. The compression of both arteries led to absence of detectable intraneural flow in all participants.

### 3.2. Assumable Dominance of Supply

Based on sonography, the superficial palmar arch was present in all individuals; no stenosis or variation in the ulnar and radial artery was found. Twenty-six individuals had absent intraneural flow during radial artery compression. Only six of those had visible flow during ulnar artery compression. Conversely, 25 participants had absent intraneural flow during ulnar artery compression; of those, only 5 had detectable flow during radial artery compression. Thus, a strong overlap existed, as a total of 20 participants showed absent flow during the compression of both radial and ulnar arteries. On the contrary, only nine individuals had maintained flow during the compression of either artery.

### 3.3. (Inverse) MAT

The full results of the MAT and Barbeau tests are shown in Table 3 and Table 4, respectively. MAT with the release of the ulnar artery (simulating a loss of the radial artery) was normal in 77.5%, intermediate in 5%, and abnormal in 17.5% of individuals. In all of those with an abnormal MAT (*n* = 7), the prior-performed radial artery compression (Table 2) had led to an absent intraneural flow, and flow had only been preserved in two during ulnar artery compression.

Inverse MAT with the release of the radial artery (simulating a loss of the ulnar artery) was rated normal in 92.5% and abnormal in 7.5%. In those with an abnormal result (*n* = 3), the absence of intraneural blood flow from prior ulnar artery compression was noted in two cases. Radial artery compression had led to absent intraneural flow in the same two individuals.

### 3.4. (Reverse) Barbeau Test

Radial artery compression did not cause a damping of the pulse wave in 53%, while in 15% the wave was lost without recovery within 2 min of testing. The results from ulnar artery compression were somewhat similar, while the temporary occlusion of both arteries caused a loss of the pulse wave without recovery in 85% of individuals, and only 5% had a persistent pulse wave.

### 3.5. Symptomatic Individuals

A total of 13 participants (32.5%) developed symptoms during arterial compression, such as pain, tingling or numbness. We refrain from assigning those to either claudication or CTS. All symptoms fully resolved after the investigation had been finalized. A comparison of symptomatic vs. asymptomatic individuals is shown in Table 5. In symptomatic individuals, intraneural flow was absent during the compression of the radial and the ulnar artery in 92.3% of cases. It was still visible in half of the asymptomatic volunteers (48.1% and 51.9%, respectively); the difference was statistically significant. Moreover, when performing the Barbeau test while compressing both arteries, the pulse wave was lost without recovery in all symptomatic cases, while it trended to be less pronounced in asymptomatic cases. When performing the MAT with a release of the radial artery, a higher rate of abnormal results was detected (*p* = 0.040), suggesting a dominant ulnar supply in those.

## 4. Discussion

This study found that simulated RAO and UAO diminish the intraneural blood flow of the median nerve at the wrist. The impairment of flow was detected in two thirds of volunteers during the compression of either vessel, despite an intact superficial palmar arch in all participants. One third of the study population became symptomatic, with the majority of those having absent intraneural flow during noninvasive occlusion testing.

### 4.1. Distal Median Nerve Blood Supply

**Figure 3:**
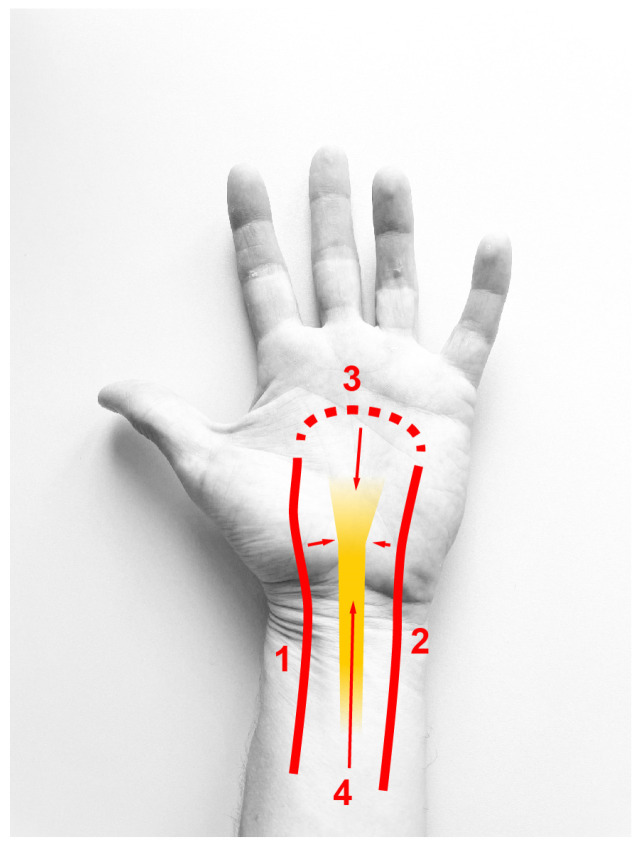
Schematic image of the blood supply to the median nerve at the wrist. Sources of blood supply: 1 = radial artery, 2 = ulnar artery, 3 = recurrent flow from palmar arches, 4 = longitudinal arterial chain.

Peripheral nerves are supplied by arteries in a segmental fashion during their course, feeding into a longitudinal arterial chain that runs within the nerve [16]. This longitudinal system maintains a perfusion of segments without direct arterial supply, such as in the carpal tunnel, where the median nerve does not receive external branches [16,17]. Instead, the median nerve at the wrist receives blood from the radial and ulnar artery in the distal forearm, and retrograde through the palmar arches. Giesen and colleagues [18] report a large radial artery branch 6 cm proximal to the radial styloid process as the main supply. Another study [16] likewise found a major radial branch at the level of the pronator quadratus. On the contrary, Blunt [19] described a constant supply from the ulnar artery just proximal to the carpal tunnel. Others found perforators from both arteries [17]. Distal to the carpal tunnel, the nerve is supplied from branches of the superficial and deep palmar arches. The imagination of an arcuate vessel anastomosing the radial and ulnar arteries is oversimplified and not present in the majority of cases. A radioulnar arch resembling this classic assumption of the superficial palmar arch is found in 36–56%, where the terminal ulnar artery anastomoses with the superficial volar branch of the radial artery. The second most prevalent type of complete superficial arch formation is represented by the terminal ulnar artery supplying all fingers including the index finger and thumb [20,21]. The completeness of the superficial arch has been reported in 43–97% of individuals and in 67–100% for deep arches [11]. Sonography revealed that a superficial arch was present in 100% of individuals in our study; however, the subtype of arch formation in terms of completeness and radial or ulnar dominance remains unclear from the ultrasound. Deep arches were not assessed in our protocol. Likewise, a systematic mapping of branches supplying the nerve is beyond what can currently be achieved through ultrasound. Based on the anatomic literature above, RAO or UAO could result in severe or virtually no consequences based on the individual’s longitudinal arterial chain and branches from radial, ulnar or palmar arch branches (Figure 3). On the contrary, radial/ulnar occlusion at the wrist could obliterate both the dominant local feeder to the nerve and a major contribution to the palmar arch.

### 4.2. Results from Occlusion Testing

Simulated RAO halted sonographic intraneural flow in 65%, and it was absent in 62.5% during UAO. These groups also strongly overlapped, with a total of 20 individuals having absent flow in both instances. Only nine individuals had maintained sonographic flow during both RAO and UAO. Not all of them developed symptoms, however, as shown in Table 5, and the observed phenomenon may be partly explainable by the ultrasound technique. Nevertheless, a correlation between sonographic and clinical findings was found in our cohort. The results of the MAT and inverse MAT were less clearly correlated, but simulated UAO led to more abnormal results in symptomatic individuals. We hypothesize that this may be due to the dominant input of the ulnar artery to the superficial palmar arch in many [20,21]. In a publication by the RADAR trial investigators [22], the ulnar blood supply was found to represent a relevant recruitable reserve capacity to the hand’s collateral circulation during impaired radial flow. This may in part explain our findings in simulated UAO with the previous manipulation of the radial artery based on our protocol.

The simulated occlusion in our study represents a short-lived event and merely serves as a proof of concept. The effects of in vivo RAO or UAO cannot be directly inferred from our findings. The prevalence of RAO decreases over time and is influenced by various patient and procedural factors [23], and there are different underlying pathophysiologic processes in acute and chronic stages of RAO [24]. Future studies should aim at investigating ongoing nerve ischemia or the eventual recruitment of vascularization in patients with acute or chronic occlusion. Nevertheless, our findings may provide further support for the use of patent hemostasis [25], maintaining neural perfusion while reducing the rate of RAO. The occurrence of hand or arm pain during patent hemostasis might indicate inadequate compression.

Introduced in its original form in 1929 [26] and modified in 1952 [9], the MAT still finds widespread application, but its usefulness is disputed as it does not reliably predict hand ischemia, and ischemic complications have been reported despite a normal MAT [11]. Interpreting the change in skin rubor is highly subjective, and test results can further be influenced by wrist flexion and extension. Further complicated by the variable cutoff values applied throughout the literature, physicians have argued against its use [27]. The Barbeau test was developed to provide objective measurements of plethysmography and pulse oximetry, overcoming the uncertainties from MAT [10]. In our study, the results of the Barbeau and reverse Barbeau tests did not differ between symptomatic and asymptomatic individuals. Overall, type C and D results were found in 15–20% during occlusion testing. These results differ markedly from the original publication [10] and a recent study reporting type C and D results in 3.2% and 3.6%, respectively [2]. This might be for various reasons, such as participant characteristics or the small sample size, although we believe it may be attributable to the chosen location of the pulse oximeter. We chose the middle finger, localized midway between the radial and ulnar artery, which differs from the original description using the thumb. In a few individuals with type D results, we repeated the Barbeau tests with the oximeter placed on the thumb, noting the persistence of the plethysmography signal. This was not systematically investigated, and we kept the results as per protocol, but this could partly explain the differing results between studies. Bartella and colleagues [28] assessed changes in hand perfusion with oximetry and noted the differences between fingers after radial occlusion, with the strongest impairment in the thumb. Another study testing collateral circulation with regard to RAO [15] found a trend towards slowed collateral flow to the thumb in the case of an incomplete superficial palmar arch. However, a deep palmar arch was always present, and blood flow to all digits was maintained.

### 4.3. Symptoms from Vascular Occlusion

Thirteen individuals (32.5%) in our study developed symptoms during the examination, potentially attributable to either claudication and/or nerve ischemia. We found absent intraneural flow in the majority of symptomatic volunteers both during simulated RAO and UAO, while it was maintained in half of the asymptomatic cohort. A systematic review analyzing outcomes after transradial access in cardiology [1] reported an incidence of 1.6% for sensory symptoms, 7.8% for postprocedural pain and 0.49% for hand disability. The rate of access-related arm pain within one day after radial cannulation was 4.5% in another study and was associated with the number of puncture attempts, duration of hemostasis compression and RAO, among others [6]. Pain is the most common postprocedural complaint overall; a fade of prevalence from 19% to 3% within one year has been reported by others [2]. The comparably high rate of symptoms in our study may result from the study protocol’s manipulation of both radial and ulnar artery in the forearm, while the majority of the literature emphasizes sequelae of transradial access. Agostoni et al. [29] reported on a series of patients undergoing cannulation of the ulnar artery after the failure of radial access, without any ischemic symptoms; however, follow-up was only until discharge, and later hand symptoms may have been overlooked. The presented study follows a purely mechanistic approach of acute vascular compression, leaving aside other (chronic) pathophysiologic factors that have been found relevant in the genesis of RAO [30] and that might also influence the occurrence of symptoms.

This study was not designed to distinguish between symptoms from claudication or transient functional impairment of the median nerve. The source of pain and discomfort (e.g., muscle, nerve, inflammatory response of vessel manipulation) cannot reliably be inferred from our findings of impaired neural blood flow, and we do not imply a causal relationship through this manuscript. To clarify whether symptoms are attributable to nerve dysfunction, a comparison of patients with CTS or median nerve neuropathy at the wrist and healthy controls should be performed. The addition of validated questionnaires for hand disability like QuickDASH [31] or the BCTQ [13] would not have been useful in this study, given the short-lived appearance of the symptoms in previously asymptomatic volunteers. Simultaneous electrodiagnostic testing could be helpful for distinguishing symptoms, as shown elsewhere [32]. Nevertheless, we used state-of-the-art ultrasound techniques to estimate intraneural blood flow, which have been validated to detect pathological changes in peripheral neuropathies like CTS [33].

### 4.4. Ultrasound Technique

Ultrasound has had a rapid technical evolution over the last decades and has become a diagnostic mainstay in peripheral nerve pathologies. In the workup of CTS, its diagnostic accuracy is nowadays even considered comparable to electrodiagnostic testing [34]. Recent technical developments allow the visualization of tissue microvascularization without the application of ultrasound contrast agents [35]. In brief, newly developed filters and algorithms are now capable of differentiating the movements of surrounding tissues from blood flow in very small blood vessels, which was not possible before. The use of recently introduced microvascular imaging technology also allows us to detect the direction of microvascular flow, which was not possible using Power Doppler Imaging [36]. Deeg et al. [37] reported an adequate visualization of nerve microvascularization in 100% of 26 individuals. Another study reported visible intraneural vascularity in 11% of healthy controls and 83% of CTS patients [38]. In our cohort of 52 screened individuals, 77% were suitable for sonographic analysis of intraneural blood flow. The others did not exhibit a sufficiently detectable flow for qualitative analysis. The intravenous application of sonographic contrast agents may have led to a better visualization and might have altered our findings during occlusion testing. Although side effects of the second-generation agents currently most widely used are extremely rare [39], application in this study’s setting was not deemed ethically justified.

The quantitative analysis of intraneural microvascular flow would also have been technically feasible in our study through a calculation of the vascular index [40]. However, given the small sample size of this study and non-detectable flow in 23% of patients, the vascular index was not chosen as a study endpoint.

### 4.5. Limitations

This study has limitations, most importantly the limited number of investigated individuals and the use of healthy volunteers. We did not enroll cardiovascular patients, and the majority of participants were female, which may not reflect this patient population. We did not enroll a comparison group with patients suffering from RAO or UAO or undergoing actual catheterization, but their inclusion is a future prospect. Arterial spasms of the radial and/or ulnar artery may have occurred from manual compression, potentially altering hemodynamics; a randomized order of which artery to compress could be used in future similar trials. Additional vessels may be present in some individuals, such as a persistent median artery, with an estimated prevalence of around 11% [41]. None were present in the extremities investigated in this study, but two individuals (5%) had persistent median arteries contralateral of the investigated side. Longer periods of observation might have shown hyperemic responses of tissues as a reaction to the vascular occlusion, maybe leading to a higher proportion with blood flow visible on ultrasound. A shortcoming of our study is the lack of standardization for the exact force and duration of vascular compression or the resting intervals between radial/ulnar compression.

A wide variety of MAT cutoff values has been used throughout the literature, and the final results could differ according to the chosen time thresholds. A state-of-the-art ultrasound system was used; however, the absence of an intraneural Doppler signal on the ultrasound may not rule out residual intraneural blood flow undetectable by sonography. Nevertheless, we aimed to provide consistent data through strict exclusion criteria and a thorough clinical and radiological screening of individuals. All investigations were performed by a senior radiologist with two decades of experience in peripheral nerve ultrasound, counterbalancing the fact that ultrasound remains a highly operator-dependent imaging modality, especially in the complex task used in this study. Despite the strong expertise of the senior author, it is noteworthy that in 23% of screened individuals the detection of sufficient intraneural blood flow was not possible.

## 5. Conclusions

Simulated RAO and UAO halted the sonographic intraneural blood flow of the median nerve in two thirds of healthy volunteers. One third of participants developed symptoms potentially attributable to claudication or neural ischemia. Larger studies are needed to optimize sonographic protocols and to identify risk factors for ischemic damage to the median nerve. Sonographic protocols for pre-interventional bedside screening could be developed, omitting the need for MAT and the Barbeau test.

## Figures and Tables

**Table 1:** Participant characteristics.

	Participants (*n* = 40)
Age (years)—mean (±SD)	37.9 (± 9.6)
Gender—*n*, (%)	
Female	29 (72.5)
Male	11 (27.5)
Side investigated—*n*, (%)	
Right	19 (47.5)
Left	21 (52.5)
Dominant hand	19 (47.5)
CSA wrist (cm^2^)—median (IQR)	0.110 (0.090 to 0.118)
CSA forearm (cm^2^)—mean (±SD)	0.072 (±0.015)
WFR—median (IQR)	1.444 (1.300 to 1.741)

Abbreviations: CSA = cross-sectional area; IQR = interquartile range; WFR = wrist-to-forearm ratio; SD = standard deviation.

**Table 2:** Sonographic findings.

	Participants (*n* = 40)
**Baseline**	
Direction of flow—*n*, (%)	
Proximal to distal (antegrade)	38 (95)
Distal to proximal (retrograde)	2 (5)
Radial artery compression	
Intraneural flow—*n*, (%)	
Visible	14 (35)
Absent	26 (65)
Direction of flow—*n*, (%)	
Proximal to distal (antegrade)	13 (92.9)
Distal to proximal (retrograde)	1 (7.1)
Reversal	1 (7.1)
Ulnar artery compression	
Intraneural flow—*n*, (%)	
Visible	15 (37.5)
Absent	25 (62.5)
Direction of flow—*n*, (%)	
Proximal to distal (antegrade)	12 (80)
Distal to proximal (retrograde)	3 (20)
Reversal	2 (13.3)
Radial + ulnar artery compression	
Intraneural flow—*n*, (%)	
Visible	0
Absent	40 (100)

**Table 3:** Modified Allen Test.

	Participants (*n* = 40)
Ulnar artery released—*n*, (%)	
Normal (0–5 s)	31 (77.5)
Intermediate (6–10 s)	2 (5)
Abnormal (>10 s)	7 (17.5)
Radial artery released—*n*, (%)	
Normal (0–5 s)	37 (92.5)
Intermediate (6–10 s)	0
Abnormal (>10 s)	3 (7.5)

**Table 4:** Barbeau test.

	Participants (*n* = 40)
Radial artery compression—*n*, (%)	
No damping of pulse wave	21 (52.5)
Damping of pulse wave	6 (15)
Loss of wave + recovery within 2 min	7 (17.5)
Loss of pulse wave without recovery	6 (15)
Ulnar artery compression—*n*, (%)	
No damping of pulse wave	20 (50)
Damping of pulse wave	5 (12.5)
Loss of wave + recovery within 2 min	7 (17.5)
Loss of pulse wave without recovery	8 (20)
Radial + ulnar compression—*n*, (%)	
No damping of pulse wave	2 (5)
Damping of pulse wave	3 (7.5)
Loss of wave + recovery within 2 min	1 (2.5)
Loss of pulse wave without recovery	34 (85)

**Table 5:** Comparison of asymptomatic vs. symptomatic individuals.

	Asymptomatic (*n* = 27)	Symptomatic (*n* = 13)	*p*
Age—mean (±SD)	37.7 (±10.1)	38.5 (±9.1)	0.932
Gender—*n*, (%)			1.000
Female	20 (74.1)	9 (69.2)	
Male	7 (25.9)	4 (30.8)	
Side investigated—*n*, (%)			0.186
Left	12 (44.4)	9 (69.2)	
Right	15 (55.6)	4 (30.8)	
Dominant hand examined—*n*, (%)	13 (48.1)	6 (46.2)	1.000
WFR—median (IQR)	1.429 (1.286 to 1.800)	1.500 (1.311 to 1.732)	0.669
Radial artery compression			
Intraneural flow—*n*, (%)			0.015
Visible	13 (48.1)	1 (7.7)	
Absent	14 (51.9)	12 (92.3)	
Barbeau test—*n*, (%)			0.253
No damping of pulse wave	14 (51.9)	7 (53.8)	
Damping of pulse wave	6 (22.2)	0	
Loss of wave + recovery within 2 min	4 (14.8)	3 (23.1)	
Loss of pulse wave without recovery	3 (11.1)	3 (23.1)	
Ulnar artery compression			
Intraneural flow—*n*, (%)			0.013
Visible	14 (51.9)	1 (7.7)	
Absent	13 (48.1)	12 (92.3)	
Reverse Barbeau test—*n*, (%)			0.116
No damping of pulse wave	15 (55.6)	5 (38.5)	
Damping of pulse wave	4 (14.8)	1 (7.6)	
Loss of wave + recovery within 2 min	2 (7.4)	5 (38.5)	
Loss of pulse wave without recovery	6 (22.2)	2 (15.4)	
Radial + ulnar artery compression			
Barbeau test—*n*, (%)			0.334
No damping of pulse wave	2 (7.4)	0	
Damping of pulse wave	3 (11.1)	0	
Loss of wave + recovery within 2 min	1 (3.7)	0	
Loss of pulse wave without recovery	21 (77.8)	13 (100.0)	
(Inverse) Modified Allen Test			
Ulnar artery released—*n*, (%)			0.242
Normal (0–5 s)	26 (96.3)	11 (84.6)	
Intermediate (6–10 s)	0	0	
Abnormal (>10 s)	1 (3.7)	2 (15.4)	
Radial artery released—*n*, (%)			0.040
Normal (0–5 s)	23 (85.2)	8 (61.5)	
Intermediate (6–10 s)	2 (7.4)	0	
Abnormal (>10 s)	2 (7.4)	5 (38.5)	

## Data Availability

The raw data supporting the conclusions of this article will be made available by the authors on request.

## Abbreviations

BCTQ	Boston Carpal Tunnel Questionnaire
CTS	Carpal tunnel syndrome
MAT	Modified Allen Test
RAO	Radial artery occlusion
UAO	Ulnar artery occlusion
WFR	Wrist-to-forearm ratio
